# The Effect of Pringle Maneuver Applied during Living Donor Hepatectomy on the Ischemia-Reperfusion Injury Observed in the Donors and Recipients

**DOI:** 10.3390/medicina60040649

**Published:** 2024-04-18

**Authors:** Yasin Dalda, Sami Akbulut, Tevfik Tolga Sahin, Adem Tuncer, Zeki Ogut, Basri Satilmis, Ozlem Dalda, Mehmet Gul, Sezai Yilmaz

**Affiliations:** 1 Department of Surgery and Liver Transplant Institute, Inonu University Faculty of Medicine, 44280 Malatya, Turkey; yasindalda@gmail.com (Y.D.); tevfiktolgaa@gmail.com (T.T.S.); ademtuncer89@hotmail.com (A.T.); sezai.yilmaz@inonu.edu.tr (S.Y.); 2 Department of Biochemistry, Inonu University Faculty of Pharmacy, 44280 Malatya, Turkey; basri.satilmis@inonu.edu.tr; 3 Department of Pathology, Inonu University Faculty of Medicne, 44280 Malatya, Turkey; ozlem.dalda@inonu.edu.tr; 4 Department of Histology and Embryology, Inonu University Faculty of Medicne, 44280 Malatya, Turkey; mehmet.gul@inonu.edu.tr

**Keywords:** liver transplantation, Pringle maneuver, ischemia–reperfusion injury, living liver donors, recipients, immune response, inflammatory response

## Abstract

*Background and Objectives*: The aim of this study is to evaluate the clinical and laboratory changes of ischemia and reperfusion injury in the remnant livers of donors with and without Pringle maneuver. Furthermore, we evaluated the recipients who have been transplanted with liver grafts from these donors. *Methods and Materials*: A total of 108 patients (54 living liver donors and 54 liver recipients) who underwent donor hepatectomy and recipients who living donor liver transplantation, were included in this randomized double-blind study between February 2021 and June 2021. The donors were divided into two groups: Pringle maneuver applied (*n* = 27) and Pringle maneuver not applied (*n* = 27). Similarly, recipients with implanted liver obtained from these donors were divided into two groups as the Pringle maneuver was performed (*n* = 27) and not performed (*n* = 27). Blood samples from donors and recipients were obtained on pre-operative, post-operative 0 h day (day of surgery), post-operative 1st day, post-operative 2nd day, post-operative 3rd day, post-operative 4th day, post-operative 5th day, and liver tissue was taken from the graft during the back table procedures. Liver function tests and complete blood count, coagulation tests, IL-1, IL-2, IL-6, TNF-α, and β-galactosidase measurements, and histopathological findings were examined. *Results*: There was no statistically significant difference in the parameters of biochemical analyses for ischemia-reperfusion injury at all periods in the donors with and without the Pringle maneuver. Similarly, there was no statistically significant difference between in the recipients in who received liver grafts harvested with and without the Pringle maneuver. There was no statistically significant difference between the two recipient groups in terms of perioperative bleeding and early bile duct complications (*p* = 0.685). In the histopathological examinations, hepatocyte damage was significantly higher in the Pringle maneuver group (*p* = 0.001). *Conclusions*: Although the histological scoring of hepatocyte damage was found to be higher in the Pringle maneuver group, the Pringle maneuver did not augment ischemia-reperfusion injury in donors and recipients that was evaluated by clinical and laboratory analyses.

## 1. Introduction

Liver transplantation is the treatment of choice in end-stage liver disease [1,2,3,4]. Shortage of deceased organ donations has resulted in the development of various strategies such as living donor liver transplantation (LDLT), use of marginal deceased donor liver grafts, and split LTs. The marginal (extended criteria) donors are especially prone to ischemia and reperfusion injury (IRI) [5,6]. IRI is inevitable in LT [7,8]. During the surgical procedure, the first step of the procedure is the procurement of the partial or full-size liver graft during which the vascular supply of the graft is temporarily interrupted creating an ischemic environment. During this period the graft is preserved in cold. In the second step, vascular anastomoses are performed, and during this period the graft temperature increases causing warm ischemia. In the last step, the graft reperfusion is performed which creates a more hostile environment augmenting the hepatocellular damage [7]. Furthermore, the liver graft can experience vascular complications that result in ischemia in the postoperative period which results in additional ischemic assaults [9,10].

During the ischemic phase of the liver graft, the molecular oxygen is reduced, and the cells switch to anaerobic metabolism resulting in a rapid drop in ATP levels [11]. The liver graft is cooled down to reduce metabolic activity and prevent tissue necrosis and injury, and this period is called the cold ischemia period. Various changes occur in the parenchymal cells that determine the level of the reperfusion injury [8]. These changes include an influx of calcium and electrolytes into the cells due to the dysfunction of ATP-dependent ion channels which results in apoptosis [12,13,14]. Furthermore, during the ischemic phase, xanthine dehydrogenase is converted to xanthine oxidase which results in over production of xanthine and free radicals (ROS) enhancing the cellular damage in the reperfusion phase when the vascular supply of the graft is reinstituted, and molecular oxygen is available [15,16,17,18]. During this phase, ROS causes organelle and DNA damage and causes the release of the damage-associated molecular patterns (DAMP) to the graft environment [19]. In the reperfusion phase, hepatocellular damage is further augmented; also, DAMPs are released into the systemic circulation. DAMPs activate the proinflammatory signal transduction pathway and the complement system. One of the pathways that is activated is the phosphoinositide 3-kinase (PI3K)/Akt (protein kinase B) [20,21]. In addition, it promotes the release of potent pro-inflammatory cytokines; Tumor Necrosis Factor-α (TNF-α), and Interleukin-6 [21]. The pro-inflammatory environment activates the components of the innate immune system and causes the attraction of the neutrophils to the ischemic site [11].

The severity and the extent of hepatic IRI determine the outcome of LT. Prolonged hepatic IRI is a risk factor for prolonged postoperative ICU stay, biliary strictures, primary non-function, reduced graft survival, and acute or chronic rejection [9,22,23,24,25,26]. Marginal liver grafts are very prone to IRI. However, IRI is equivocally important in LDLT due to the reduced functional liver mass in LDLT [9].

One of the factors that might contribute to IRI during LDLT is using the Pringle maneuver during donor hepatectomy (LDH). Pringle maneuver was first described in 1906 by Dr. James Hogarth Pringle and was used to control the hepatic inflow by clamping the hepatoduodenal ligament. The main goal is to decrease the amount of bleeding during major hepatectomies. Studies have shown that it can be safely performed for up to 60 min during major hepatic surgery to decrease the bleeding from the liver and major vessels during the surgery and to prevent adverse outcomes and complications in the postoperative period [27,28,29,30]. In addition, it has been shown that Pringle maneuver could be protective against ischemic damage; a phenomenon called ischemic preconditioning [31,32,33,34]. In LDLT, the data regarding the effect of Pringle maneuver applied during LDH on the IRI in the recipients is lacking.

The primary aim of this study was to evaluate the effect of pringle maneuver in living liver donors (LLDs) during the postoperative period. The secondary aim was to evaluate the clinical implications of IRI in recipients who received living donor liver grafts that were procured with and without Pringle maneuver during LDH. The data obtained from this study will be useful to define the effects of Pringle maneuver, performed under necessary conditions during LDH, on the postoperative outcomes of both LLDs and the recipients.

## 2. Materials and Methods

This is a prospective, double-blind, randomized study conducted on LLDs and LT recipient patients who undergoing surgery for elective LDLT at Inonu University Liver Transplant Institute between February and June 2021. The power analysis and sample size of this study were calculated according to the alanine aminotransferase (ALT) values reported in the study by Park and colleagues [power (1-β) = 90%, α = 0.05, tails = two, allocation ratio = 1, total sample size = 74] [35] First, considering the main logic of this study, LLDs were divided into two groups: those with and without pringle maneuver during LDH procedure, and an equal number of LLDs were assigned to each group using the simple randomization method. LLD candidates who met universal donor evaluation criteria and were willing to participate were included in this study. Then, the LT recipients were similarly divided into two independent groups based on whether pringle maneuver was applied to the thier LLDs. Recipients under 18 years of age, emergency procedures, and transplantations from deceased donor organs were not included in the study. Two groups of LLDs who underwent LDH with and without Pringle maneuver were compared among themselves. Similarly, the recipients into whom the liver graft obtained from the mentioned LLDs were implanted were also compared among themselves. Thus, in this study, the effect of the pringle maneuver on both LLDs and their recipients was evaluated within the same study design, but completely independently of each other. The study was approved by the institutional review board of the Malatya Clinical Research Ethics Committee (approval date: 18 November 2020 and number: 178). The study was funded by the Scientific Research Project Coordination Unit of Inonu University (Grant Number: TSA-2021-2390).

### 2.1. Surgical Technique for LDH Procedure

All procedures were performed in our institute by the surgical team as routine procedures. We have previously published our donor hepatectomy technique and all procedures are performed according to principles previously defined [36,37]. Pringle maneuver was decided according to simple randomization technique (drawing lots) and the donor surgeons were informed on the day of the operation. Pringle maneuver was applied for 10 min by clamping the hepatoduodenal ligament as the parenchymal transection was started during donor hepatectomy. Parenchymal transection was performed by using Cavitron Ultrasonic Surgical Aspirator (CUSA^®^ Excel; Integra LifeSciences, Princeton, NJ, USA). All grafts were flushed with chilled Ringer’s Lactate and Histidine Tryptophan Ketoglutarate (HTK) solution during the “Back-Table” procedures and necessary preparations for implantation were performed. At the end of the “Back-Table” procedure, liver biopsy that was 1 cm in diameter was taken from all the grafts.

### 2.2. Study Parameters

Our aim is to evaluate the clinical implications of IRI in both recipients and donors. Therefore, we have collected all the demographic variables including age, gender, height, weight, body mass index (BMI), blood groups, and the relation between the donor-recipient groups for all the participants of the study. Clinical characteristics including the indication of transplantation, Model for End Stage Liver Disease (MELD) scores, graft weight, warm and cold ischemia durations, the type of living donor liver grafts (left versus right lobe grafts), and the remnant liver volumes were recorded for all the participants.

Blood samples were collected on preoperative (PreOP), immediate postoperative (POD0; day of surgery), POD 1st, 2nd, 3rd, 4th and 5th days. Our routine laboratory values (that are obtained from every donor and recipient in the preoperative and postoperative period) include hemoglobin (g/dL), hematocrit (%), Platelet count (corpuscle/mm^3^), White blood cell counts (cells/mm^3^), International normalized ratio (INR), Blood urea nitrogen (BUN) (mg/dL), Creatinine (Cr) (mg/dL), albumin (Alb) (g/dL), total bilirubin (T. Bilirubin) (mg/dL), direct bilirubin (D. Bilirubin) (mg/dL), aspartate amino transferase (AST) (IU/mL), alanine amino transferase ALT (IU/mL), alkaline phosphatase (ALP) (IU/mL), γ-glutamyl transferase (GGT) (IU/mL), lactate dehydrogenase (LDH) (IU/mL), and ammonia (mmol/L). We obtained these tests for all the participants at the designated intervals stated above and defined them as routine laboratory tests. This was in our routine donor follow-up protocol as it has been reported earlier [36,37]. Therefore, the data was obtained from the electronic patient database of our institution.

We evaluated specific laboratory parameters related to IRI including interleukin-1 (IL-1), interleukin-2 (IL-2), interleukin-6 (IL-6), tumor necrosis factor-α (TNF-α), β-Galactosidase (β-Gal). All specific laboratory parameters were analyzed in Inonu University Liver Transplant Institute Hepatology research laboratories. Blood samples were collected from all the participants on PreOp, POD 1st, 2nd, and 3rd intervals, and specific laboratory parameters were studied at each time interval. These parameters were measured using the Enzyme-Linked immunosorbent Assay (ELISA) kit. Human IL-1 ELISA kit (Catalog number: E0077Hu, Bioassay Technologies Laboratory, Shanghai, China), human IL-2 ELISA kit (Catalog number: E0094Hu, Bioassay Technologies Laboratory, Shanghai, China), human IL-6 ELISA kit (Catalog number: E0090Hu, Bioassay Technologies Laboratory, Shanghai, China), human TNF-α ELISA kit (Catalog number: E0082Hu, Bioassay Technologies Laboratory, Shanghai, China), and human β-Gal ELISA kit (Catalog number: E0856Hu, Bioassay Technologies Laboratory, Shanghai, China) were used for the analysis. The analyses were performed according to the protocol provided by the manufacturer. Briefly, all the reagents were warmed to room temperature before the analyses. The standard (positive control) was diluted to the recommended dilutions. Fifty µL of the standards were added to the designated wells provided by the manufacturer. Forty µL of the samples were added to the wells and 10 µL of primary antibody was added on to each sample. Fifty µL of streptavidin-horse radish peroxidase solution was added to each well and the wells were incubated at 37 °C for 60 min. At the end of the incubation period, all the wells were washed five times with wash buffer. Substrates A and B were added to each well at a volume of 50 µL to each well and incubated at 37 °C for 10 min. Fifty µL of stop solution was added to each well. Absorbency was measured at 450 nm using BioTek Synergy H1m (BioTek, Winooski, VT, USA) microplate reader, and the concentrations of the evaluated parameters were calculated.

### 2.3. Histologic Evaluation

The liver tissues obtained from the liver grafts during the “Back-Table” procedures were fixed in 10% Formalin for 48 h. The liver tissues were exposed to different concentrations of xylene. In the next step, the tissues were dehydrated using increasing concentrations of ethanol. The tissues were embedded in paraffin at 62 °C. Six micrometer tissue sections were prepared and stained with Hematoxylin and Eosin (H&E). The sections were analyzed using Nikon Eclipse Ni-U light microscope, images were captured using the Nikon DS-Fi3 microscope camera, and the image analyses were performed using Nikon NIS-Elements Documentation 5.02 image analysis software (Nikon Corporation, Tokyo, Japan). In H&E-stained liver tissues, hepatocyte damage was evaluated and scored according to the presence of cytoplasmic eosinophilic staining, pyknosis, karyolysis, hydropic degeneration, hepatocyte necrosis, vacuolization. Portal and parenchymal inflammatory cell infiltration were scored between 0 and 3 (0: none, 1: weak, 2: moderate, 3: strong) [38].

### 2.4. Statistical Analysis

The normal distribution of the continuous variables was analyzed using Kolmogorov-Smirnov test. Majority of the continuous variables did not distribute normally, and for this reason, continuous variables were expressed as the median and interquartile range (IQR). Qualitative variables were expressed as number of affected individuals and the percentage of the study population (%). The quantitative variables were compared using the Mann-Whitney U test between the study groups. Categoric variables were compared between the study groups using the Chi-Square test. Any *p*-value < 0.05 was considered as statistically significant. All statistical analyses were performed using the Statistical Software Package for Social Sciences version 25.0 (SPSS v25.0) (IBM Corp, Armonk, NY, USA).

## 3. Results

### 3.1. Demographic and Clinical Characteristics of the LLDs

There were 54 donors with ages ranging from 18 to 41 years (median: 28 years IQR: 13). Thirty-four donors were male (63%) and 20 were female (37%). The BMI of the donors ranged between 17.3 to 32.6 kg/m^2^ (median: 23.8 kg/m^2^ and the IQR: 5.6). Fourty-seven (87%) of the grafts were right lobe grafts and 7 (13%) were left lobe grafts. Median remnant liver volumes were 31% (IQR: 2) and ranged between 29% and 35%. The distribution of the blood group was 0 Rh(+) in 24 (44.4%), A Rh(+) in 16 (29.9%), B Rh(+) in 9 (16.7%), 0 Rh(−) in 4 (4.74%), and AB Rh(+) in 1 (1.9%) of the donors. Eleven of the donors (20.4%) were non-related to their recipient and 43 (79.6%) were 1st to 4th-degree relatives of their recipients. All the demographic, clinical, and operative characteristics of the donors are summarized in Table 1.

### 3.2. Evaluation of the LLDS with and without the Pringle Maneuver

The demographic, clinical, and operative characteristics of the donor did not significantly change among the study groups. Furthermore routine laboratory parameters obtained on PreOP, POD0, POD 1st, POD 2nd, POD 3rd, POD 4th, POD 5th days did not significantly differ between the donors with and without pringle maneuver (Table 1). The specific laboratory parameters including IL-1, IL-2, IL-6, TNF-α, and β-galactosidase did not significantly differ between the study groups at any interval (PreOP, POD0, POD 1st, POD 2nd, POD 3rd days) (Table 2).

### 3.3. Demographic and Clinical Characteristics of the Recipients

The median age of the recipients was 54 years (IQR: 23) and ranged between 19 to 71 years. Thirty-four (63%) recipients were male and 20 (37%) were female. The median body mass index was 26 kg/m^2^ (IQR: 5.6) and ranged between 17.8 to 41.6 kg/m^2^. The distribution of the blood groups was A Rh(+) in 22 (40.7%), 0 Rh(+) in 15 (27.8%), B Rh(+) in 10 (18.5%), AB Rh(−) in 4 (7.4%), and 0 Rh(−) in 3 (5.6%) of the recipients. The etiologies of the end-stage-liver failure were Hepatitis B (HBV) related liver disease in 11 (20.4%), hepatocellular carcinoma (HCC) in 7 (13%), Budd-Chiari in 6 (11.1%), HBV + HCC in 10 (18.5%)I, other various reasons such as non-alcoholic steatohepatitis (NASH), autoimmune hepatitis, primary sclerosing cholangitis, primary biliary cirrhosis, Wilsons disease, and alcoholic cirrhosis in 10 (18.5%) of the patients The MELD-Na scores of the patients ranged between 15 to 39 (median: 18 and IQR: 7). The graft weights ranged between 450 gr to 1040 gr (median: 738 and IQR: 199). Median cold ischemia time (CIT) was 94 min (IQR: 4) and ranged between 36 to 301 min. Warm ischemia times (WIT) ranged between 12 to 115 min (median: 51 and IQR: 23). The details of the demographic and clinical data of the recipients are summarized in Table 3.

### 3.4. Evaluation of the Recipients That Received Liver Grafts with or without Pringle Maneuver

The demographic, clinical, and operative characteristics of the recipients did not significantly differ among the study groups. The routine laboratory parameters obtained on PreOP, POD0, POD 1st, POD 2nd, POD 3rd, POD 4th, POD 5th did not show significant difference between the study groups (Table 3). Also, the specific laboratory parameters (IL-1, IL-2, IL-6, TNF-α, and β-galactosidase) obtained on PreOP, POD0, POD 1st, POD 2nd, POD 3rd days did not show statistically significant differences among the recipients that received liver grafts with or without Pringle maneuver during donor hepatectomy (Table 4).

IRI in liver transplantation can cause ischemic-type biliary complications and biliary strictures [39]. We evaluated biliary complications in recipients in the postoperative 1st month. Five patients (18.5%) recipients who received grafts with the Pringle maneuver developed biliary complications (stricture or leak). On the other hand, 5 patients (18.5%) who received grafts without the Pringle maneuver developed biliary stricture or leak. The two groups did not show any statistical difference in terms of biliary complications (*p* = 0.685).

### 3.5. Microscopic Effects of the Pringle Maneuver

**Figure 1:**
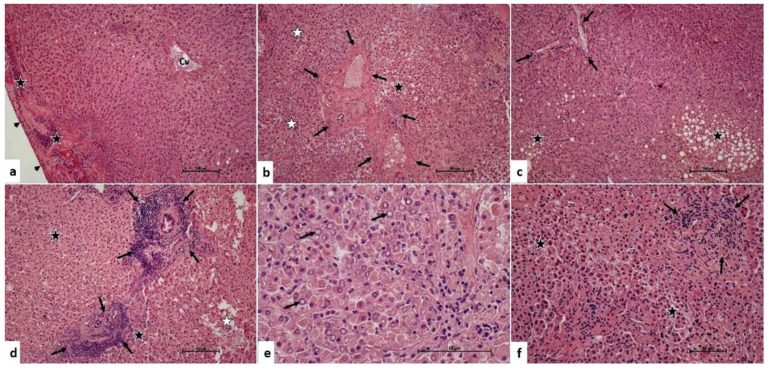
Pringle maneuver not applied group. (**a**) Central venule (Cv), inflammatory cell infiltration in the subcapsular area (star), Glisson’s capsule (arrowhead). H-E, ×10 (**b**). Portal area (arrows), hydropic damage and degeneration of hepatocytes (black star), hepatocytes with heterochromatic-pycnotic nuclei (white star). H-E, ×10 (**c**). Portal area (arrows), hydropic damage, and degeneration of hepatocytes (star). H-E, ×10 (**d**). Dense inflammatory cell infiltration in portal areas (arrows), hepatocytes with heterochromatic-pycnotic nuclei (black star), hepatocyte degeneration (white star). H-E, ×10 (**e**). Chromatolysis (arrow) in hepatocyte nuclei. H-E, ×40. (**f**). Inflammatory cell infiltration in the portal area (arrows), hepatocytes with eosinophilic cytoplasm, heterochromatic-pycnotic nuclei (star). H-E, ×10.

In liver grafts harvested without the Pringle maneuver, we observed that there was a mild inflammatory cell infiltration in the portal and parenchymal areas. In addition, there was intracytoplasmic vacuolization and bile pigment accumulation in the hepatocytes. There were hepatocytes with eosinophilic cytoplasm and heterochromatic pyknotic nuclei. Some areas even showed chromatolysis in the hepatocyte nuclei. There were focal areas of hydropic changes and hydropic degeneration (Figure 1). The median inflammatory cell infiltration score of these grafts was 1 (IQR: 0). The median hepatocyte damage score was 1 (IQR: 1).

**Figure 2:**
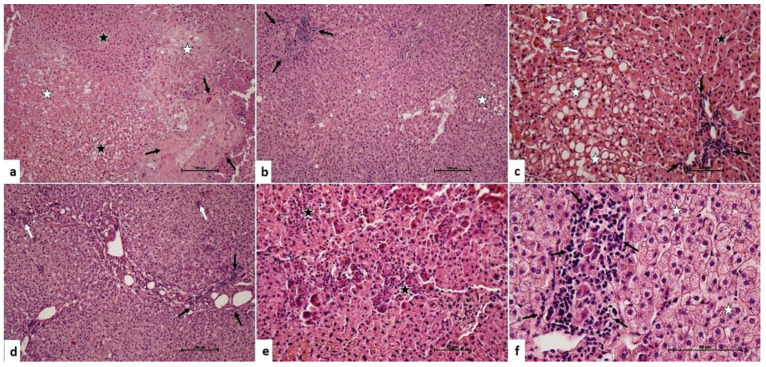
Pringle maneuver applied group. (**a**) Portal area (arrows), hepatocytes with heterochromatic-pycnotic nuclei (black star), hepatocyte degeneration (white star). H-E, ×10 (**b**). Inflammatory cell infiltration in the portal area (arrows), intracytoplasmic vacuolization in hepatocytes (star). H-E, ×10 (**c**). Inflammatory cell infiltration in the portal area (black arrows), intracytoplasmic bile pigmentation in hepatocytes (white arrows), hepatocytes with heterochromatic-pycnotic nuclei (black star), intracytoplasmic vacuolization in hepatocytes (white star). H-E, ×20 (**d**). Inflammatory cell infiltration in the portal area (black arrows), inflammatory cell infiltration in focal areas in the liver parenchyma (white arrows). H-E, ×10 (**e**). Granulomatous hepatocyte degeneration (star). H-E, ×20 (**f**). Inflammatory cell infiltration in the portal area (arrows), hydropic changes in hepatocytes (star). H-E, ×40.

In the liver grafts harvested with the Pringle maneuver, there were a similar type of microscopic changes. However, the severity and the extent of the damage was higher than the liver grafts without the Pringle maneuver (Figure 2). The median inflammatory cell infiltration score was 1 (IQR: 0). The median hepatocyte damage score was 2 (IQR: 1).

None of the livers in the study groups showed periportal edema. The liver damage score of the liver grafts with the Pringle maneuver was statistically higher than the liver grafts without the Pringle maneuver (*p* = 0.001). The results of the histopathological analyses of the livers in the study groups are summarized in Table 5.

## 4. Discussion

The liver transplantation technique has evolved extensively through the last 3 decades [1]. Liver transplantation is the gold-standard treatment of acute and chronic end-stage liver disease, various liver tumors, metabolic liver disease, and parasitic diseases such as alveolar hydatic cysts [1,40,41]. The demand for liver grafts exceeds the available deceased donor organ supply. This has led to the development of techniques such as LDLT. LDLT has been a widely accepted form of liver graft supply in Asian countries and Turkey where deceased donor organ supply is limited [1,42]. Together with the advancements in immunosuppressive drugs, surgical techniques, and postoperative intensive care, patient survival and quality of life have increased significantly in liver transplant recipients [43].

Donor safety is of paramount importance in LDLT. The most important complication in donor hepatectomy is bleeding. The pringle maneuver is used to temporarily interrupt the inflow of the liver to control parenchymal bleeding [28]. It is used by many surgeons during major liver resections and major hepatic trauma. The Pringle maneuver involves clamping the hepatoduodenal ligament reducing the flow through the portal vein and the hepatic artery. This technique naturally induced hepatic IRI which limits its use in many transplant centers [44]. On the other hand, various studies show the beneficial effects of the Pringle maneuver on IRI in sustained hepatic ischemia-reperfusion episodes of the liver [31,32]. Murry and colleagues [33] defined ischemic preconditioning as brief periods of ischemia and reperfusion episodes that protect the tissues from chronic and sustained ischemic periods. The results of our study showed that the Pringle maneuver did not affect the inflammatory cytokines in both donors and recipients. Furthermore, we did not observe any functional alterations due to the Pringle maneuver.

Ischemia and reperfusion injury is a dynamic process that involves cellular and humoral immune response, and free oxygen and nitrogen radicals. In liver transplantation, severe IRI is a major factor causing postoperative graft dysfunction [45,46,47]. Kupffer cells, sinusoidal endothelial cells, neutrophils, thrombocytes, and many complex molecular pathways have a role in hepatic IRI in liver transplantation [9]. It was shown that hepatic IRI in liver transplantation caused biliary problems, prolongation of postoperative hospitalization duration and various other complications such as rejection, and postoperative acute kidney injury [25,48]. In the present study, we did not observe any change in renal function in any of the donors or the recipients. Furthermore, we did not observe any biliary problems in any recipients. We only performed the Pringle maneuver for 10 min during the parenchymal transection period which may not cause any significant changes in the liver grafts.

Park and colleagues [35] have conducted a similar study as ours on 50 living donors and have shown that the Pringle maneuver (25 donors) has resulted in higher serum ALT levels. The bleeding was lower in the donors with the Pringle maneuver. They have also analyzed the serum levels of inflammatory and regenerative cytokines such as IL-6, IL-8, TNF-α, and hepatocyte growth factor (HGF) and found no difference between the two groups. Takatsuki and colleagues [49] analyzed the effect of the Pringle maneuver during living donor hepatectomy and reported lesser bleeding without any hepatic functional compromise in the Pringle group. Our results showed that liver function tests and biomarkers of hepatocyte damage did not significantly change with performing the donor hepatectomy with the Pringle maneuver.

There have been various studies regarding the effect of the pringle maneuver application during various surgical procedures such as liver resection for malignancy and donor hepatectomy; the results of which showed that the amount of blood loss was reduced and there were no significant effects on the remnant liver functions [32,44,50,51]. In our study, we found that the Pringle maneuver could safely be applied to donors without compromising liver functions. The most striking finding of our study was that the Pringle maneuver was performed in donors who have less than 30% future remnant.

Determination of hepatic IRI is difficult. The morphologic findings of hepatic IRI have been reported as eosinophilia, pyknosis, chromatin fragmentation, steatosis, and vacuolization [52,53]. Delourdes and colleagues [53] have reported that histopathological changes associated with 20 min of hepatic IRI animal models were completely reversed in the postoperative 28th day. The current research on hepatic IRI is performed on experimental models that performed ischemia between 15 to 60 min [52,53,54,55,56]. In our study, we have performed histopathologic analyses on the liver grafts harvested with and without the Pringle maneuver and liver specimens were obtained during the “Back-Table” procedures. We have shown that although we have performed only 10 min of the Pringle maneuver, the hepatocyte damage scores were higher in the group with the Pringle maneuver. Although there was no significant impact on the liver function tests, we believe that these changes were reversed following the implantation and reperfusion of the liver grafts. Our study is the first study to show the morphological effects of hepatic IRI in humans.

We have performed a randomized prospective study evaluating the clinical implications of hepatic IRI caused by the Pringle maneuver performed during donor hepatectomy. However, the present study has some limitations. The most important limitation is the short duration of the Pringle maneuver that was applied. Furthermore, we have not performed any analyses on the long-term results of the Pringle maneuver. Also, ischemic preconditioning provided by the Pringle maneuver has not been analyzed. In addition, although we have met our goal regarding our sample size, there are many uncontrollable conditions during research on human subjects such as the medications that are performed in the intensive care unit and the in-patient wards which may have hampered our results. Therefore, we believe a greater sample size will provide more accurate results in future research.

Despite all the limitations, we have shown that even if the Pringle maneuver is applied for 10 min, it results in histopathologic changes in the early perioperative period without any functional implications for the donor and the recipient. The effects may be more prominent with prolonged application durations. Therefore, we believe that the Pringle maneuver can be safely performed in selected cases if the interval is kept short. Further studies are required to evaluate the long-term effects of the Pringle maneuver. Also, studies evaluating the effects of the Pringle maneuver on ischemic preconditioning should be evaluated. All these studies will provide the necessary information to apply the Pringle maneuver routinely or selectively.

## Figures and Tables

**Table 1:** The demographic, operative, and biochemical characteristics of the living liver donors.

Donor Characteristics	Pringle Maneuver Applied (*n* = 27)	Pringle Maneuver Not Applied (*n* = 27)	*p*
Age	29 (15)	28 (14)	0.165
BMI	24.5 (5.6)	23.8 (6.1)	0.716
Future Remnant Liver Volume (%)	32 (3)	30 (2)	0.319
Gender [*n* (%)]			
Male	18 (67)	16 (59)	
Female	9 (33)	11 (41)	
Liver graft [*n* (%)]			
Right lobe	23 (85.2)	24 (88.8)	
Left lob	4 (14.8)	3 (11.1)	
WBC (PreOP)	7.4 (2.1)	7.4 (1.8)	0.653
WBC (POD5)	7 (3.1)	7.3 (2.4)	0.489
HB (PreOP)	15.4 (3.1)	14.9 (2.7)	0.574
HB (POD5)	12.1 (3.1)	11.9 (2.1)	0.640
PLT (PreOP)	272 (106)	265 (75)	0.959
PLT (POD5)	206 (96)	225 (44)	0.299
INR (PreOP)	0.9 (0.1)	0.9 (0.09)	0.748
INR (POD5)	1 (0.1)	1.1 (0.1)	0.373
Albumin (PreOP)	4.5 (0.3)	4.5 (0.5)	0.689
Albumin (POD5)	3.3 (0.2)	3.4 (0.4)	0.274
T. Bilirubin (PreOP)	0.5 (0.2)	0.5 (0.3)	0.800
T. Bilirubin (POD5)	1.1 (0.8)	1 (0.7)	0.267
D. Bilirubin (PreOP)	0.1 (0.07)	0.1 (0.09)	0.869
D. Bilirubin (POD5)	0.3 (0.3)	0.3 (0.3)	0.710
AST (PreOP)	18 (6)	19 (8)	0.482
AST (POD5)	59 (26)	63 (36)	0.986
ALT (PreOP)	16 (7)	16 (9)	0.537
ALT (POD5)	106 (78)	115 (57)	0.809
ALP PreOP	70 (27)	67 (30)	0.842
ALP (POD5)	81 (51)	89 (40)	0.993
GGT (PreOP)	13 (36)	17 (8)	0.222
GGT (POD5)	44 (72)	50 (69)	0.406
LDH (PreOP)	184 (30)	171 (49)	0.287
LDH (POD5)	242 (75)	295 (119)	0.213

BMI: Body mass index, PreOP: Preoperative days, POD: Postoperative days, WBC: White blood cell, HB: Hemoglobin, PLT: platelet, INR: international normalized ratio, T. Bilirubin: Total bilirubin, D. Bilirubin: Direct bilirubin, AST: Aspartate amino transferase, ALT: Alanine amino transferase, ALP: Alkaline phosphatase, GGT: γ-glutamyl transferase, LDH: Lactate dehydrogenase. Quantitative variables are given as median (IQR). The quantitative variables were compared using the Mann-Whitney U test between the study groups. Categoric variables were compared between the study groups using the Chi-Square test. Normal value ranges of the routine laboratory parameters that are analyzed in our study are as follows: WBC [4.3–10.2 × 10^3^ corpuscles/µL], HB [13.6–17.2 g/dL], PLT [156–373 × 10^3^ corpuscles/µL], INR, Albumin [3.5–5 g/dL], T. Bilirubin [0.2–1.2 mg/dL], D. Bilirubin [0–0.5 mg/dL], AST [0–35 U/L], ALT [0–55 U/L], ALP [34–104 U/L], GGT [9–64 IU/L], LDH [125–243 U/L].

**Table 2:** The specific laboratory parameters including the serum levels of inflammatory cytokines and biomarkers of liver damage in donors who received living donor hepatectomy with and without the Pringle maneuver.

Donor Characteristics [Median (IQR)]	Pringle Maneuver Applied (*n* = 27)	Pringle Maneuver Not Applied (*n* = 27)	*p*
IL-1 (PreOP)	42 (135)	40 (89)	0.424
IL-1 (POD0)	42 (134)	51 (74)	0.735
IL-1 (POD1)	43 (136)	46 (92)	0.531
IL-1 (POD2)	47 (132)	46 (66)	0.788
IL-1 (POD3)	52 (132)	48 (73)	0.309
IL-2 (PreOP)	290 (1016)	330 (651)	0.780
IL-2 (POD0)	281 (1045)	334 (448)	0.754
IL-2 (POD1)	286 (939)	338 (637)	0.842
IL-2 (POD2)	289 (1009)	300 (556)	0.910
IL-2 (POD3)	304 (1018)	318 (564)	0.689
IL-6 (PreOP)	92 (262)	100 (194)	0.682
IL-6 (POD0)	111 (251)	104 (183)	0.429
IL-6 (POD1)	102 (261)	106 (176)	0.828
IL-6 (POD2)	95 (235)	104 (178)	0.958
IL-6 (POD3)	98 (254)	100 (115)	0.544
TNF-α (PreOP)	132 (323)	107 (255)	0.677
TNF-α (POD0)	116 (352)	120 (197)	0.795
TNF-α (POD1)	127 (258)	116 (221)	0.965
TNF-α (POD2)	135 (333)	123 (256)	0.795
TNF-α (POD3)	122 (413)	123 (170)	0.476
B-gal (PreOP)	132 (404)	162 (399)	0.806
B-gal (POD0)	136 (387)	176 (286)	0.965
B-gal (POD1)	174 (389)	159 (213)	0.965
B-gal (POD2)	165 (375)	168 (323)	0.917
B-gal (POD3)	152 (383)	154 (229)	0.896

PreOP: preoperative days, POD: postoperative days, IL-1: interleukin-1, IL-2: interleukin-2, IL-6: interleukin-6, TNF-α: tumor necrosis factor-α, B-gal: β-Galactosidase. Quantitative variables are given as median (IQR). The quantitative variables were compared using the Mann-Whitney U test between the study groups. Categoric variables were compared between the study groups using the Chi-Square test.

**Table 3:** The demographic, clinical, operative, and biochemical characteristics of the recipients.

Recipient Characteristics [Median (IQR)]	Pringle Maneuver Applied (*n* = 27)	Pringle Maneuver Not Applied (*n* = 27)	*p*
Age	54 (23)	53 (23)	0.959
BMI	27.1 (6)	25.1 (5.5)	0.545
Gender [*n* (%)]			0.259
Male	15 (56)	19 (70)	
Female	12 (44)	8 (30)	
MELD	20 (7)	18 (7)	0.631
Graft weight (gr)	700 (270)	760 (178)	0.736
Cold ischemia time (min)	93 (40)	95 (49)	0.634
Warm ischemia time (min)	47 (25)	54 (20)	0.121
WBC PreOP	3.9 (2.1)	5 (2.4)	0.054
WBC POD5	5.4 (5.6)	6.8 (4.2)	0.528
Hb PreOP	9.9 (2.4)	11.1 (4.4)	0.100
Hb POD5	8.8 (1.2)	8.8 (1.5)	0.729
Plt PreOP	86 (72)	103 (73)	0.104
Plt POD5	52 (49)	56 (42)	0.279
INR PreOP	1.5 (0.4)	1.4 (0.3)	0.775
INR POD5	1.4 (0.4)	1.3 (0.3)	0.337
Alb PreOP	3 (1.5)	2.8 (0.9)	0.430
Alb POD5	3.2 (0.5)	3.3 (0.6)	0.238
T. Bilirubin PreOP	2.1 (4.1)	2.3 (5.5)	0.171
T. Bilirubin POD5	3.9 (3.6)	3.1 (4.3)	0.421
D. Bilirubin PreOP	0.6 (2.3)	0.8 (2.9)	0.177
D. Bilirubin POD5	1.8 (3)	1.7 (2.9)	0.324
AST PreOP	42 (35)	71 (86)	0.059
AST POD5	64 (25)	52 (47)	0.368
ALT PreOP	29 (25)	39 (31)	0.019
ALT POD5	161 (146)	136 (75)	0.137
ALP PreOP	124 (77)	141 (101)	0.406
ALP POD5	76 (50)	72 (40)	0.672
GGT PreOP	47 (84)	85 (71)	0.113
GGT POD5	104 (156)	104 (136)	0.742
LDH PreOP	220 (95)	258 (105)	0.071
LDH POD5	217 (109)	221 (94)	0.789
Ammonia PreOP	160	171	0.513
Ammonia POD 5	87 (48)	89 (65)	0.606

BMI: body mass index, PreOP: preoperative, POD: postoperative, WBC: white blood cell, Hb: hemoglobin, Plt: platelet, INR: international normalized ratio, Alb: albumin, T. Bilirubin: total bilirubin, D. Bilirubin: direct bilirubin, AST: aspartate amino transferase, ALT: alanine amino transferase, ALP: alkaline phosphatase, GGT: γ-glutamyl transferase, LDH: lactate dehydrogenase. The quantitative variables were compared using the Mann-Whitney U test between the study groups. Categoric variables were compared between the study groups using the Chi-Square test. Normal value ranges of the routine laboratory parameters that are analyzed in our study are as follows: WBC [4.3–10.2 × 10^3^ corpuscles/µL], HB [13.6–17.2 g/dL], PLT [156–373 × 10^3^ corpuscles/µL], INR, Albumin [3.5–5 g/dL], T. Bilirubin [0.2–1.2 mg/dL], D. Bilirubin [0–0.5 mg/dL], AST [0–35 U/L], ALT [0–55 U/L], ALP [34–104 U/L], GGT [9–64 IU/L], LDH [125–243 U/L].

**Table 4:** The specific biochemical parameters including the serum levels of inflammatory cytokines and biomarkers of liver damage in recipients who received liver grafts with and without the Pringle maneuver.

Recipient Characteristics [Median (IQR)]	Pringle Maneuver Applied (*n* = 27)	Pringle Maneuver Not Applied (*n* = 27)	*p*
IL-1 PreOP	39 (63)	39 (58)	0.577
IL-1 POD0	56 (123)	48 (45)	0.112
IL-1 POD1	60 (123)	53 (34)	0.152
IL-1 POD2	68 (125)	57 (44)	0.339
IL-1 POD3	65 (126)	55 (36)	0.249
IL-2 PreOP	280 (307)	245 (503)	0.573
IL-2 POD0	376 (992)	366 (502)	0.340
IL-2 POD1	368 (958)	384 (319)	0.331
IL-2 POD2	498 (932)	447 (508)	0.434
IL-2 POD3	480 (984)	380 (386)	0.657
IL-6 PreOP	93 (84)	79 (100)	0.331
IL-6 POD0	112 (249)	101 (88)	0.224
IL-6 POD1	114 (245)	98 (77)	0.129
IL-6 POD2	119 (247)	103 (96)	0.384
IL-6 POD3	116 (248)	109 (76)	0.440
TNF-a PreOP	106 (170)	94 (138)	0.390
TNF-a POD0	169 (408)	111 (191)	0.132
TNF-a POD1	158 (394)	128 (101)	0.087
TNF-a POD2	170 (396)	158 (135)	0.708
TNF-a POD3	193 (402)	135 (140)	0.215
B-gal PreOP	140 (174)	118 (135)	0.242
B-gal POD0	199 (382)	144 (231)	0.138
B-gal POD1	206 (373)	164 (173)	0.150
B-gal POD2	185 (370)	203 (207)	0.348
B-gal POD3	245 (388)	196 (132)	0.366

PreOP: preoperative day, POD: postoperative days, IL-1: interleukin-1, IL-2: interleukin-2, IL-6: interleukin-6, TNF-a: tumor necrosis factor-α, B-gal: β-Galactosidase. The quantitative variables were compared using the Mann-Whitney U test between the study groups. Categoric variables were compared between the study groups using the Chi-Square test.

**Table 5:** The summary of the histopathological characteristics of the liver grafts with and without the Pringle maneuver.

Histopathological Scores [Ortanca (IQR)]	Pringle Maneuver Applied (*n* = 25)	Pringle Maneuver Not Applied (*n* = 26)	*p*
Inflammatory cell infiltration	1 (0)	1 (0)	0.334
Hepatocyte damage	2 (1)	1 (1)	0.001

## Data Availability

The datasets analyzed during the current study are available from the corresponding author on reasonable request.
